# ESD-VesNet: uncertainty-aware vessel segmentation network for endoscopic submucosal dissection with hard negative mining

**DOI:** 10.1007/s11548-026-03703-2

**Published:** 2026-07-03

**Authors:** Mengya Xu, Ming Chen, Zhen Li, Chaoyang Lyu, An Wang, Rulin Zhou, Chuanhao Zhao, Jiaxun Xiang, Tsz Chun Wong, Hossein Farahnaki, Sobhan Zamani Kiasari, Tong Wu, Zimeng Su, Yile Zeng, Ruijing Wen, Xiaohan Shang, Yi Mu, Kezhen Lin, Yidong Zhang, Hongliang Ren

**Affiliations:** 1 Department of Computer Science and Engineering, CUHK, Hong Kong, China; 2 Department of Electronic Engineering, CUHK, Hong Kong, China; 3 Shenzhen Research Institute, CUHK, Shenzhen, China; 4 Shenzhen Loop Area Institute, Guangdong, China; 5 Department of Gastroenterology, Qilu Hospital of Shandong University, Jinan, China

**Keywords:** Endoscopic submucosal dissection, Vessel segmentation, SAM3, Evidential deep learning, Uncertainty estimation

## Abstract

**Purpose:**

Preventing intraoperative bleeding in endoscopic submucosal dissection (ESD) relies on the timely identification and pre-coagulation of submucosal vessels. We aim to reduce bleeding risk by delivering accurate vessel segmentation with uncertainty awareness.

**Methods:**

We present ESD-VesNet, a prompt-free SAM3-based framework with an evidential head that outputs vessel probability and pixel-wise uncertainty. The model is trained in a false-positive-aware manner using our developed ESD-Vessel dataset, which includes 2401 positives (images with clear vessel annotation) and 708 curated hard negatives (images may be confused with vessels), combined with online uncertainty-guided hard negative mining. The model is trained in a false-positive-aware manner using the ESD-Vessel dataset, which includes 2401 positive samples (images with clear vessel annotations) and 708 curated hard negatives (images visually confusable with vessels), combined with online uncertainty-guided hard negative mining. By suppressing false positive predictions, our approach has the potential to minimize unnecessary interventions, such as unwarranted coagulation.

**Results:**

On the validation set, ESD-VesNet achieves Dice$_{FG}$ 52.11%, IoU$_{FG}$ 35.23%, mDice 75.70%, and Vessel Detection Rate 96.81%, while keeping Background False Positive Rate at 0.61%. Structural quality is high (E-measure 0.7275; S-measure 0.7223). Qualitative analyses show robustness under bubbles, debris, reflections, and blood seepage.

**Conclusion:**

Integrating SAM3 with evidential uncertainty and hard negative mining yields a clinically oriented vessel segmentation system that maintains high detection sensitivity with low false positives, supporting safer ESD.

## Introduction

Endoscopic submucosal dissection (ESD) is a minimally invasive treatment option for early-stage gastrointestinal cancers, offering advantages that include reduced surgical trauma and rapid postoperative recovery. However, the ESD procedure is highly complex, particularly due to the intricate vascular structure within the submucosal layer, which poses significant safety challenges during dissection. When using an electrosurgical knife for tissue dissection, failure to detect concealed vessels beforehand can lead to direct vascular rupture due to mechanical cutting and electrothermal effects, triggering intraoperative bleeding. As the primary complication of endoscopic submucosal dissection (ESD), intraoperative bleeding not only obscures the surgical field, requiring repeated irrigation to locate the source, but may also prolong the procedure or necessitate conversion to open surgery if not properly managed, thereby significantly increasing patient risk.

Currently, most research on intraoperative bleeding focuses on post-bleeding management, such as bleeding detection and dynamic bleeding source localization [1–5]. In contrast, we emphasize bleeding prevention through early warnings for vascular zones and prompt intervention. To prevent bleeding, clinicians perform pre-coagulation on potential vessels using electrosurgical knives or hemostatic forceps before dissection. However, pre-coagulation heavily relies on the clinician’s proficiency in accurately identifying vessels. Studies indicate that approximately 20% of intraoperative adverse events stem from human visual perception errors [6], including misjudgments of anatomical structures. Without computational assistance, endoscopists achieve an average vessel detection rate (VDR) of only about 56.4% [7]. Accurate vessel segmentation also helps identify vascular zones as high-risk areas during the dissection phase, minimizing unintended contact and reducing bleeding risk. Therefore, AI-assisted real-time detection and segmentation of vessels holds immense clinical value for improving procedural safety.

Despite significant advances in computer vision for medical image segmentation, vessel segmentation in ESD environments remains highly challenging. First, the textures and colors of submucosal vessels are highly similar to surrounding mucosal tissue with indistinct boundaries, making precise edge detection difficult for traditional algorithms. Second, ESD surgical environments are highly dynamic. The presence of bubbles, tissue debris, smoke, and minor blood seepage during dissection creates severe visual occlusions and image artifacts, frequently triggering algorithmic false negatives or false positives. These challenges necessitate specialized approaches that can handle both the morphological subtlety of vessels and the noise characteristics of the ESD environment.

To address these challenges, this work proposes ESD-VesNet, a vessel segmentation framework for ESD based on the Segment Anything Model 3 (SAM3) [8] foundation model with uncertainty-aware learning. The main contributions of this work include:

- We introduce the ESD-Vessel dataset, comprising 44 high-definition surgical procedures with 2401 annotated vessel frames and 708 hard negative frames. To the best of our knowledge, this is the first ESD-vessel dataset that explicitly incorporates negative samples to suppress false positives during training.
- We propose ESD-VesNet, a framework designed for accurate vessel detection and segmentation in ESD procedures. By integrating evidential deep learning (EDL) for uncertainty quantification with hard negative mining, our method achieves superior segmentation quality while maintaining high vessel detection rates and low false-positive rates.

## Related works

### Surgical vessel dataset

High-quality annotated datasets constitute the foundation for advancing surgical artificial intelligence systems. Most publicly available surgical anatomy datasets, including CholecSeg8k and LapGC-KVAD-30 [9], originate from laparoscopic procedures. These datasets predominantly feature external surgical approaches targeting large and medium-sized perigastric vessels. In contrast, ESD operates within the confined submucosal layer, where vessels exhibit substantially different morphological characteristics, size distributions, and visual appearances compared to their laparoscopic counterparts. The unique imaging conditions in endoscopic environments, characterized by close-range illumination, specular reflections, and limited depth perception, further distinguish ESD-vessel imaging from laparoscopic visualization.

Existing ESD-vessel segmentation research has predominantly relied on limited-scale private datasets. As summarized in Table 2, prior studies by Ebigbo et al. [10] and Yang et al. [11] employed datasets with relatively low resolutions (256×256 to 512×640) and modest sample sizes (1000–2000 frames from 16–30 procedures). These dataset limitations, particularly the absence of hard negative samples and insufficient coverage of diverse surgical scenarios, constrain model generalization and increase susceptibility to false-positive predictions in challenging intraoperative conditions.

### Segmentation models

Deep learning has seen considerable development in vascular segmentation. Early research focused on retinal vascular segmentation, where U-Net and its highly automated variant nnU-Net, demonstrated outstanding performance in capturing the intricate vascular structures of the eye. Ebigbo et al. [10] achieved real-time clinical detection of intraoperative vessels during ESD using DeepLabv3+ combined with a ResNet-50 backbone, significantly improving physician VDR. Subsequently, Yang et al. [11] enhanced ESD segmentation quality by incorporating reward-optimized uncertainty estimation based on an improved TransUNet architecture.

The SAM series models [8, 12] demonstrate powerful zero-shot image segmentation capabilities. Medical SAM 2 [13] further address the limitations of general models in medical applications by extending SAM2’s performance to medical video and 3D vascular segmentation. The SAM2-Adapter [14] study achieves improved performance in occluded object detection through parameter-efficient fine-tuning (PEFT). For the specific anatomical structure of blood vessels, VesSAM [15] further optimizes SAM to reduce missed detections of extremely thin, highly curved vessels. The latest SAM3 introduces a presence head to pre-detect target existence before segmentation, reducing false positives.

## Methods

### ESD-vessel dataset

**Fig. 1:**
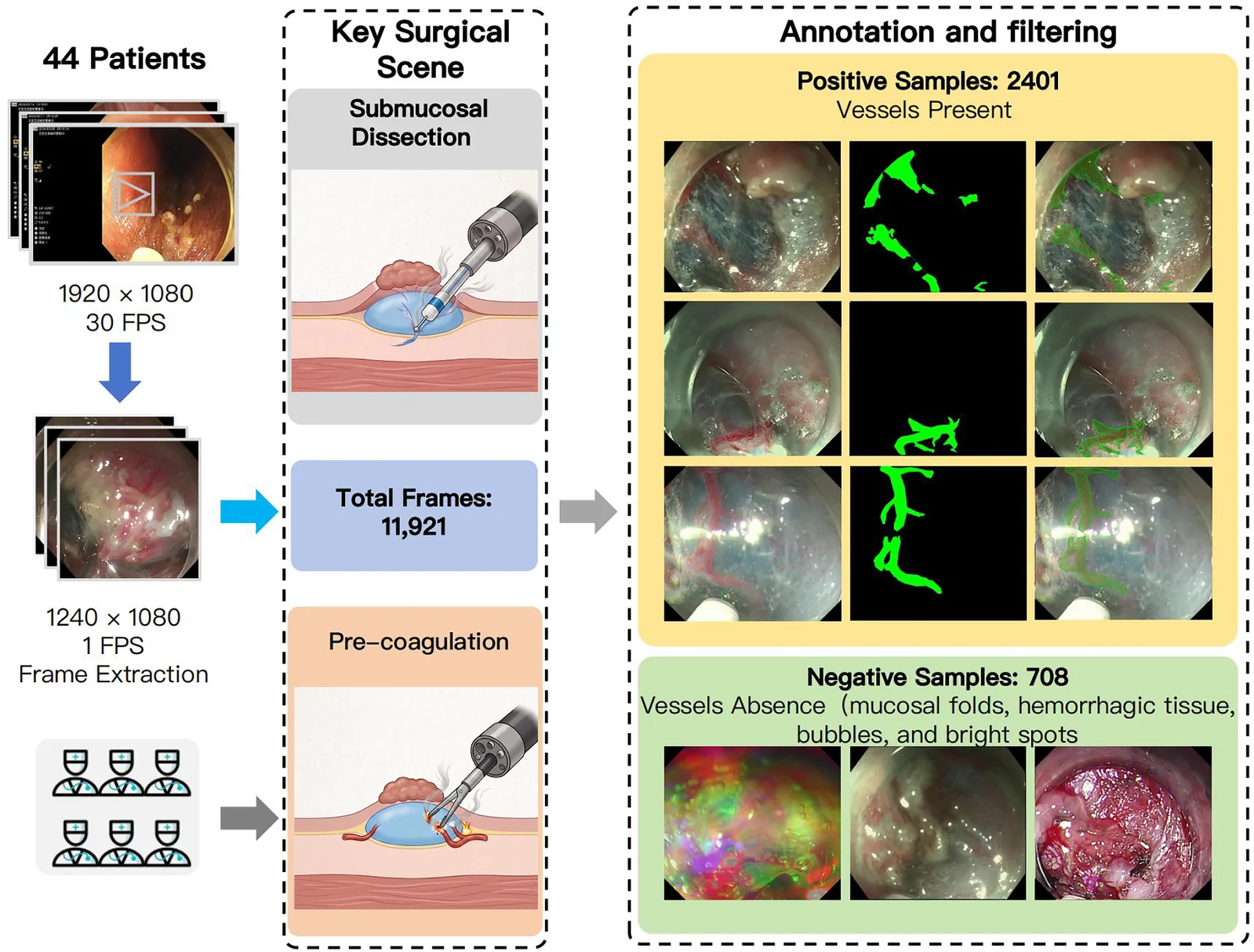
Overview of the ESD-Vessel dataset. The dataset comprises 3109 annotated frames from 44 high-definition ESD procedures, including positive samples with vessel annotations and hard negative samples containing confusable structures such as mucosal folds, hemorrhagic regions, and specular highlights

**Fig. 2:**
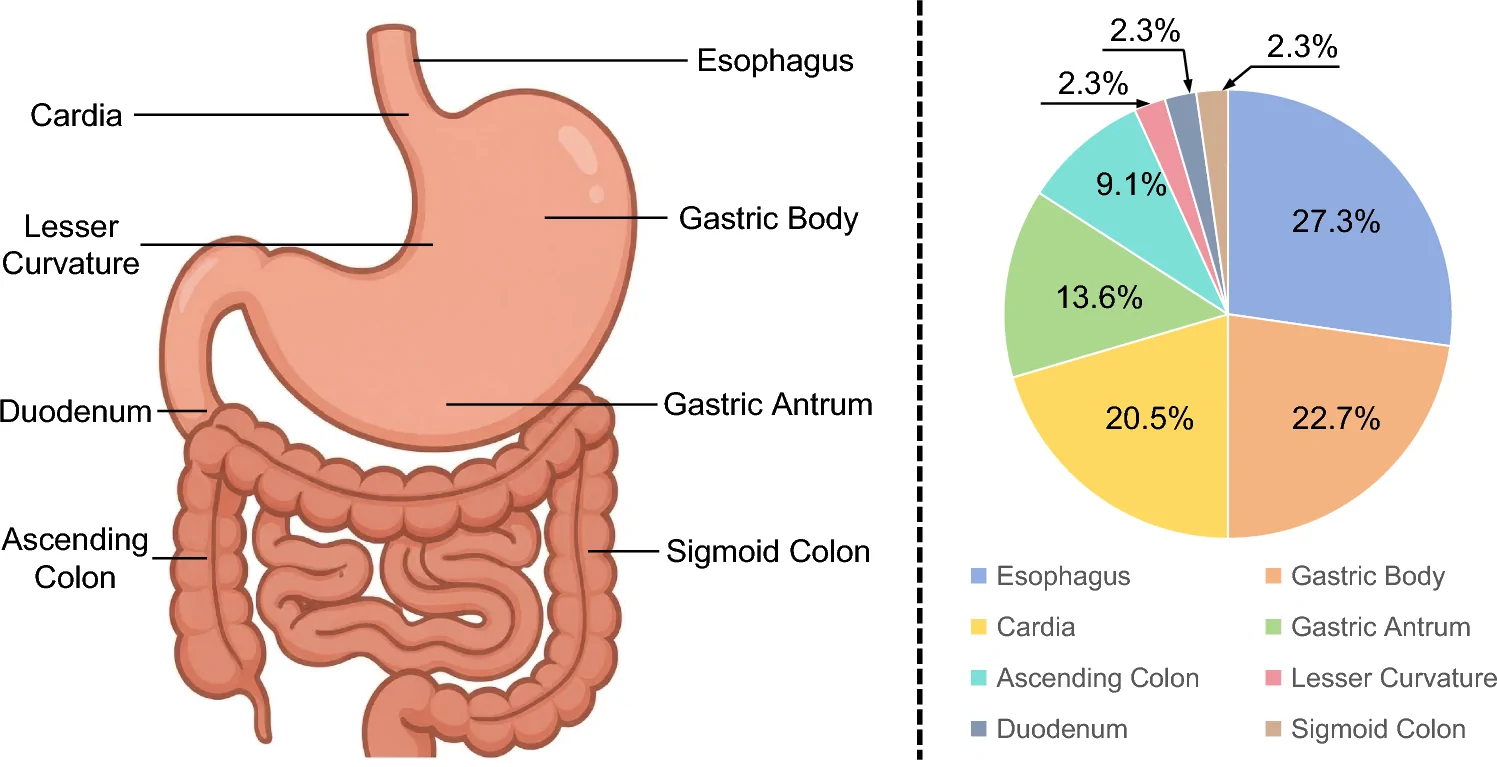
Distribution of the 44 source ESD videos across 8 anatomical sites. The left panel shows the percentage of videos from each site, and the right panel illustrates the corresponding anatomical locations

As shown in Fig. 1, the dataset was constructed from **44** high-definition ESD videos collected at Qilu Hospital of Shandong University. These source videos cover 8 anatomical sites, including Esophagus, Cardia, Gastric Body, Lesser Curvature, Gastric Antrum, Duodenum, Sigmoid Colon, and Ascending Colon. The site distribution of the raw video cohort is summarized in Fig. 2. All data were fully anonymized. The surgical videos were recorded at 30 FPS with a resolution of 1920×1080. We cropped the images to 1240×1080, retaining the complete surgical field of view.

Surgical clips were specifically selected from the pre-coagulation and submucosal dissection phases, as these phases provide the most frequent and clear exposure of submucosal vascular structures. These clips were extracted as key frames at a sampling rate of 1 FPS, totaling 11921 frames.

Endoscopy specialists from Qilu Hospital identified images containing distinct and clear vessels, which were annotated as positive samples using the LabelMe tool. Additionally, we curated hard negative frames, which are images devoid of vessels yet visually confusable with them (e.g., mucosal folds, hemorrhagic regions, air bubbles, and specular highlights), to suppress false-positive predictions.

Our final **ESD-Vessel** dataset comprises **3109** frames, including **2401** positive frames with vessel annotations and **708** hard negative frames. Although the raw video cohort covered 8 anatomical sites, the final frame-level dataset covered 7 sites, because the *Lesser Curvature* videos did not yield frames suitable for reliable vessel annotation. The frame-level distribution of the final dataset is summarized in Table 1. The data were partitioned at the patient level into a training set of 2764 frames (2060 positives, 704 negatives) and a validation set of 345 frames, ensuring no patient overlap between subsets.

**Table 1:** Frame-level distribution of the final ESD-vessel dataset across anatomical sites

Anatomical site	Total samples	Positive	Negative	Surgical stage
Gastric body	1465	1167	298	Submucosal Dissection, Pre-coagulation
Esophagus	1278	906	372	Submucosal Dissection, Pre-coagulation
Sigmoid colon	140	140	0	Submucosal Dissection, Pre-coagulation
Gastric antrum	101	85	16	Submucosal Dissection, Pre-coagulation
Ascending colon	50	46	4	Submucosal Dissection, Pre-coagulation
Duodenum	45	33	12	Submucosal Dissection
Cardia	30	24	6	Pre-coagulation
Total	3109	2401	708	

### ESD-VesNet architecture

Given an endoscopic frame $I\in \mathbb {R}^{H\times W\times 3}$, our goal is to predict a vessel probability map $\hat{G}\in [0,1]^{H\times W}$ and a binary mask $\bar{G}\in \{0,1\}^{H\times W}$. The ground-truth vessel mask is denoted as $G\in \{0,1\}^{H\times W}$ (vessel=1, background=0).

**Fig. 3:**
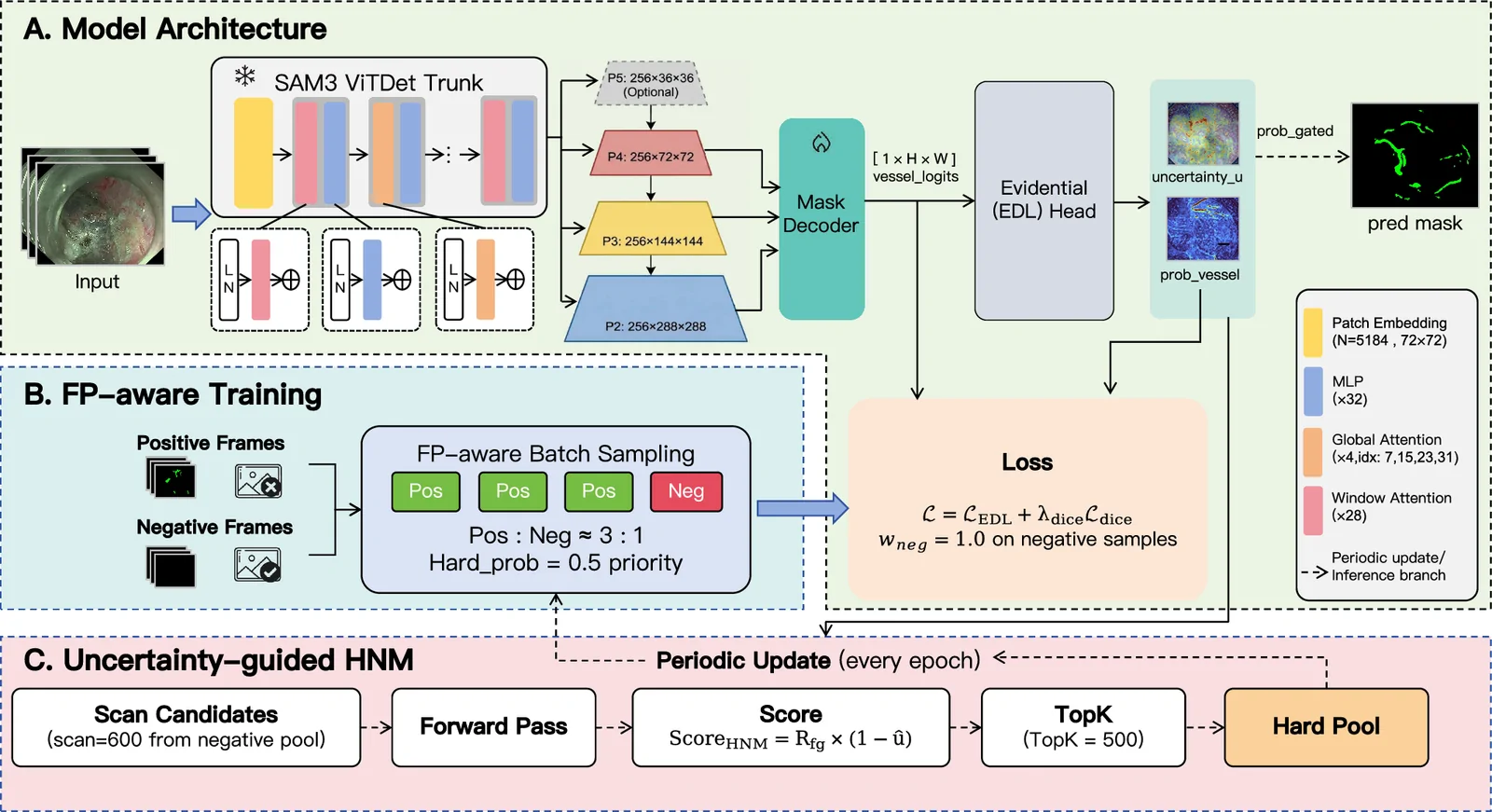
Overview of the proposed framework. The architecture integrates three key modules: (i) SAM3 ViTDet encoder (Trunk + Neck) that extracts multi-scale features (P2–P5), (ii) prompt-free SAM decoder that generates vessel logits without interactive prompts, and (iii) evidential deep learning (EDL) head that outputs vessel probability, pixel-wise uncertainty, and gated probability for robust false-positive suppression

Our framework (see Fig. 3) consists of three modules: (i) a SAM3 ViTDet image encoder (Trunk+Neck), (ii) a prompt-free SAM decoder (Prompt Encoder + Mask Decoder) that produces vessel_logits, and (iii) an evidential (EDL) head that outputs prob_vessel, uncertainty_u, and prob_gated in a single forward pass. For clarity, we use mathematical symbols in equations while keeping a one-to-one correspondence with the implementation: $\texttt {vessel\_logits}\leftrightarrow Z$, $\texttt {prob\_vessel}\leftrightarrow p_{\textrm{vessel}}$, $\texttt {uncertainty\_u}\leftrightarrow u$, and $\texttt {prob\_gated}\leftrightarrow p_{\textrm{gated}}$.

#### SAM3 ViTDet trunk and neck

To satisfy SAM3 Rotary Positional Embedding (RoPE) and patch alignment, the model internally resizes the input to a core resolution of 1008×1008 with patch size p=14, forming a 72×72 token grid (i.e., N=5184 patch tokens). The trunk applies M=32 Transformer blocks with a standard Pre-LN residual design and a mixture of window attention and occasional global attention blocks. The SAM3 neck converts trunk features into a multi-scale feature pyramid $\{P2, P3, P4, P5\}$ with unified channel dimension 256 (Fig. 3A). In our implementation, *P*4 is used as the main image embedding for mask decoding, while higher-resolution features (e.g., *P*2 and *P*3) provide guidance for detail recovery.

#### Prompt-free SAM decoding

Although we do not use interactive prompts (points/boxes/text), the SAM decoder is structurally conditioned on prompt embeddings. We therefore adopt a prompt-free setting by feeding empty prompts to the prompt encoder, where the dense prompt corresponds to the default no-mask embedding. The mask decoder predicts a low-resolution mask logit and bilinearly upsamples it back to the original resolution, yielding the logit map $Z\in \mathbb {R}^{1\times H\times W}$.

#### Evidential head and uncertainty gating

We apply evidential learning to obtain both calibrated vessel probability and epistemic uncertainty. Specifically, we construct a K=2 class logit vector and map it to Dirichlet parameters $\boldsymbol{\alpha }$, from which we obtain the vessel probability $p_{\textrm{vessel}}$ and the uncertainty $u=\frac{K}{\sum _{k=1}^{K}\alpha _k}$. To suppress false positives in uncertain regions, we introduce an uncertainty-gated probability (the key design of our method):

1 $$\begin{aligned} p_{\textrm{gated}} = p_{\textrm{vessel}}\cdot (1-u)^{\gamma }. \end{aligned}$$

### FP-aware training

We partition training frames into (i) positive frames with vessel annotations and (ii) negative frames that contain no vessels but are prone to false positives (e.g., folds, bubbles, specular highlights, smoke, and blood-like textures). Training uses FP-aware mini-batch construction with a fixed positive/negative ratio, and negatives are preferentially drawn from a dynamically maintained hard pool (Sect. Uncertainty-guided hard negative mining).

#### Training objective

Let the one-hot target be $\boldsymbol{y}=[1-G,\,G]$. The loss combines an EDL objective (ACE with an annealed KL regularizer) and an auxiliary soft Dice constraint on $p_{\textrm{vessel}}$ to stabilize thin-structure learning. We omit the standard ACE/KL formulae for brevity and provide the final objective as

2 $$\begin{aligned} \mathcal {L}=\mathcal {L}_{\textrm{ACE}}+\lambda (t)\mathcal {L}_{\textrm{KL}}+\lambda _{\textrm{dice}}\mathcal {L}_{\textrm{Dice}}, \end{aligned}$$

where λ(t) is an annealing schedule and $\lambda _{\textrm{dice}}$ controls the contribution of the Dice term.

**Table 2:** Comprehensive comparison of existing surgical vascular segmentation datasets (LC: Laparoscopic cholecystectomy, LDG: Laparoscopic distal gastrectomy)

Dataset	Year	Surgery type	Videos	Frames	Resolution	Availability
CholecSeg8k	2020	LC	17	8080	854×480	Public
Ebigbo et al. [10]	2022	ESD	16	2012	512×640	Private
Yang et al. [11]	2023	ESD	30	1203	256×256	Private
LapGC-KVAD-30 [9]	2025	LDG	30	5303	1920×1080	Public
**Ours (ESD-Vessel Dataset)**	**2026**	**ESD**	**44**	**3109**	**1240** × **1080**	**Private**

### Uncertainty-guided hard negative mining

Fixed-ratio negative sampling may not cover the most challenging false-positive patterns. We therefore propose an online hard negative mining (HNM) strategy guided by uncertainty. At each update, we sample a candidate subset $\mathcal {C}$ from the negative pool and run a forward pass to obtain $p_{\textrm{vessel}}(x)$ and *u*(*x*) for each candidate $x\in \mathcal {C}$. We quantify the hardness by combining (i) the predicted foreground area ratio under threshold θ and (ii) the confidence term $(1-\hat{u}(x))$, where $\hat{u}(x)$ is the spatial mean of the uncertainty map. The final hardness score is

3 $$\begin{aligned} \textrm{Score}_{\textrm{HNM}}(x)=R_{\textrm{fg}}(x)\cdot (1-\hat{u}(x)). \end{aligned}$$

We then update the hard pool by selecting the Top-*K* candidates ranked by $\textrm{Score}_{\textrm{HNM}}(x)$, and couple this pool with FP-aware sampling in subsequent training.

**Fig. 4:**
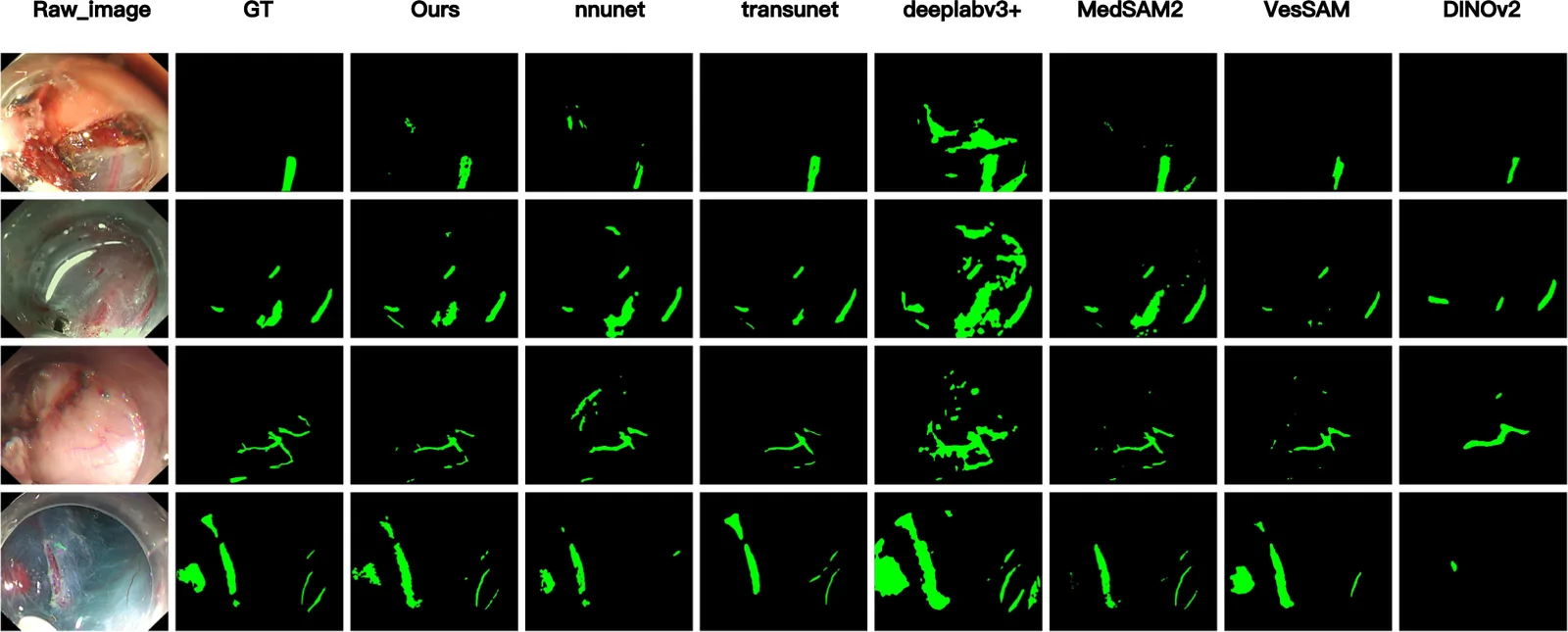
Qualitative comparison of vessel segmentation results across challenging ESD scenarios. Each row demonstrates performance under different conditions: clear vessel exposure (Row 1), partial occlusions from bubbles and tissue debris (Row 2), and extreme interference from specular reflections and blood seepage (Rows 3-4). Columns show ground-truth annotations and predictions from U-Net, DeepLabV3+, TransUNet, SAM variants, and our proposed method. Our framework demonstrates superior false-positive suppression in confusable regions while maintaining sensitivity to genuine vascular structures

### Implementation details

All experiments were implemented in PyTorch 2.4.0 and trained with DistributedDataParallel on 3× NVIDIA GeForce RTX 3090 GPUs (24GB each). CUDA version is 12.1. We used a patient-wise split with validation patients {P10, P16, P17, P25, P35, P38} and trained for 100 epochs. Input frames were resized to 1024×1024 (with an internal aligned crop of 1008×1008 for the SAM3 image encoder). The learning rate was set to $2\times 10^{-4}$ with weight decay 0.0, and automatic mixed precision was enabled for efficiency. To suppress false positives, unlabeled negative frames mined from the ELEC subset were included with ratio 0.21 (capped at 500) and a fixed negative ratio of 0.25 in each mini-batch. Hard negative mining maintained a pool of 500 samples and refreshed it every epoch by scanning 600 candidates, with hard negatives sampled with probability 0.5. For evidential learning, we used $\lambda _{\textrm{KL}}=0.01$ with anneal steps 5000, and the auxiliary Dice loss weight was set to $\lambda _{\textrm{dice}}=5.0$. Uncertainty gating was applied as $p_{\textrm{gated}}=p_{\textrm{raw}}(1-u)^{\gamma }$ with γ=1.0.For loss, we reported $\textrm{Dice}_{\textrm{FG}}$, $m\textrm{Dice}$, $\textrm{IoU}_{\textrm{FG}}$, $m\textrm{IoU}$, $\textrm{Prec}_{\textrm{FG}}$, $\textrm{BG}_{\textrm{FPR}}$, $\textrm{VDR}$ [10] ($A_{\min }$ = 1), *S*-measure, *E*-measure, and $\textrm{MAE}$ [16].

## Results

### Quantitative comparison

As shown in Table 3, we compare our method with several representative segmentation baselines on the validation set using segmentation accuracy, false-positive control, and structural quality metrics.

Given a probability map *p* (raw or gated), we binarize it with threshold θ as $\bar{G}=\textbf{1}(p>\theta )$. We report $\textrm{Dice}_{\textrm{FG}}$ and $\textrm{IoU}_{\textrm{FG}}$ computed from the global confusion matrix of $\bar{G}$ and *G*.

To rigorously evaluate the model’s ability to suppress false positives, we employ two distinct metrics. First, Background False Positive Rate, defined as $\textrm{BG}_{\textrm{FPR}}=\frac{\textrm{FP}}{\textrm{FP}+\textrm{TN}}$, specifically measures the rate of false positives occurring in background areas, reflecting the model’s resistance to environmental noise such as bubbles or smoke. Second, Foreground Precision, calculated as $\textrm{Prec}_{\textrm{FG}} = \frac{\textrm{TP}}{\textrm{TP} + \textrm{FP}}$, reflects the reliability of the predicted vessel regions. A higher $\textrm{Prec}_{\textrm{FG}}$ indicates a lower rate of vessel misdetection within the detection results, ensuring that the identified targets are genuinely vessels rather than others. We also report Vessel Detection Rate (VDR) as an image-level recall metric: for each ground-truth positive frame $x\in \mathcal {V}$, it is counted as detected if the true-positive area satisfies $\textrm{TP}(x)\ge A_{\min }$ under threshold θ, and $\textrm{VDR}$ is the fraction of detected frames in $\mathcal {V}$. To assess structural integrity, we employ S-measure ($S_{\alpha }$), E-measure ($E_{\phi }$), and Mean Absolute Error (MAE).

DeepLabV3+ achieves the highest vessel detection rate (97.39%), demonstrating strong sensitivity. However, this comes at the cost of elevated false positives ($\textrm{BG}_{\textrm{FPR}}: 8.23\%$) and poor segmentation precision (Prec$_{FG}$: 12.34%), indicating substantial over-segmentation. nnU-Net significantly reduces false positives (1.27%) but suffers from a lower detection rate (83.58%), failing to strike an optimal balance for clinical safety.

TransUNet serves as a highly competitive baseline, achieving strong segmentation results (Dice$_{FG}$: 50.36%, mDice: 74.83%). It effectively balances sensitivity and specificity. However, our proposed **ESD-VesNet framework outperforms TransUNet across all key dimensions**. Specifically, our method achieves the highest segmentation accuracy (Dice$_{FG}$: **52.11%**, mDice: **75.70%**) and intersection over union ($\textrm{IoU}_{\textrm{FG}}$: **35.23%**).

Crucially, our method demonstrates superior clinical reliability. It attains a VDR of 96.81% (ranking second only to the over-segmenting DeepLabV3+) while maintaining a low $\textrm{BG}_{\textrm{FPR}}$ of 0.61%, comparable to the most conservative models. Furthermore, ESD-VesNet achieves the best performance in structural and alignment metrics (E$_{\phi }$: **0**.**7223**), indicating that our model not only segments more vessel pixels but also better preserves the topological structure and boundary details of the vasculature.

The results validate that our design, combining the SAM3 with uncertainty-guided evidential learning successfully and hard negative mining suppresses background noise without sacrificing the sensitivity required to detect fine, camouflaged vessels.

**Fig. 5:**
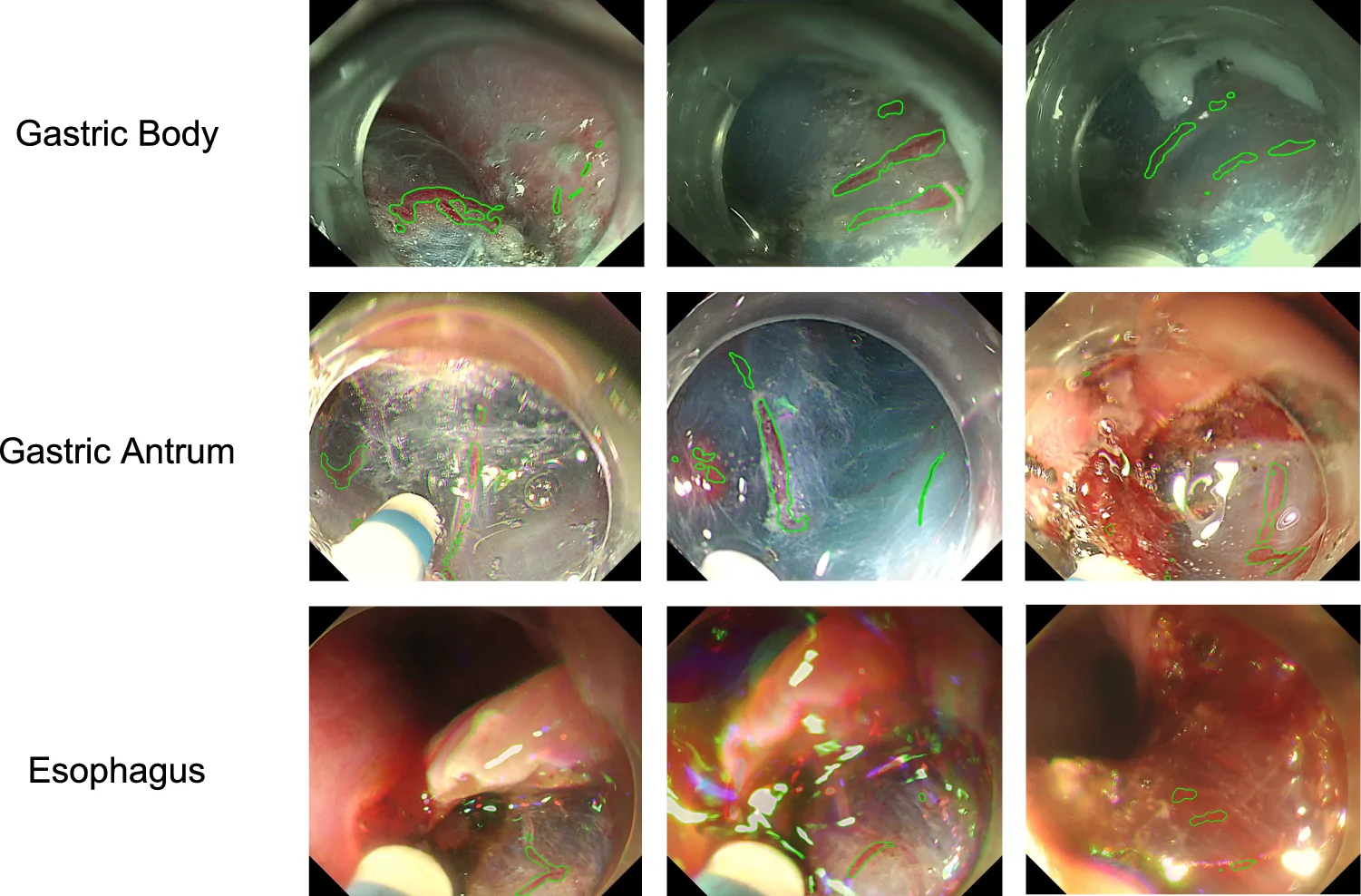
Representative vessel segmentation results on the validation set from three anatomical sites: Gastric Body, Gastric Antrum, and Esophagus. Green contours indicate the predicted vessel regions

To further assess anatomical-site generalization, we also report site-wise validation performance in Table 4. Since the validation split contains samples from only three anatomical sites, namely Gastric Antrum, Esophagus, and Gastric Body, the site-wise quantitative analysis is restricted to these three sites. Figure 5 provides representative qualitative samples from the validation set.

**Table 3:** Quantitative comparison of vessel segmentation performance on the validation set

Methods	Segmentation accuracy (%)					False positive control (%)		Structural & Alignment quality		
	Dice$_{FG}$ ↑	mDice ↑	IoU$_{FG}$ ↑	mIoU ↑	Prec$_{FG}$ ↑	BG$_{FPR}$ ↓	VDR ↑	$S_{\alpha }$ ↑	$E_{\phi }$ ↑	MAE ↓
DeepLabV3+ [10]	21.56	58.59	12.08	51.83	12.34	8.23	**97**.**39**	0.1626	0.2984	0.0919
nnU-Net [17]	36.00	67.49	21.95	59.96	31.20	1.27	83.58	0.6424	0.6774	0.0246
TransUNet [18]	*50.36*	*74.83*	*33.65*	*66.13*	*56.60*	*0.54*	85.92	0.6428	*0.6871*	0.0252
DINOv2 [19]	32.99	66.11	19.75	59.10	27.95	0.99	79.71	0.7119	0.3627	0.0173
MedSAM2 [13]	40.53	69.87	25.42	61.92	42.01	0.74	79.13	0.6863	0.3806	0.0290
VesSAM [15]	39.09	69.19	24.29	61.44	**59**.**15**	**0**.**32**	63.19	*0.7238*	0.6140	**0**.**0147**
**Ours (ESD-VesNet)**	**52**.**11**	**75**.**70**	**35**.**23**	**66**.**92**	55.71	0.61	*96.81*	**0**.**7275**	**0**.**7223**	*0.0157*

Bold indicates the best performance, and *Italics* indicates the second-best results. The symbols ↑ and ↓ indicate that higher or lower values are better, respectively

**Table 4:** Site-wise vessel segmentation performance on the validation set.

Sites	# Images	Dice$_{FG}$	mDice	IoU$_{FG}$	mIoU	S-measure	E-measure	MAE ↓
Gastric antrum	46	0.4930	0.7429	0.3272	0.6564	0.7132	0.7841	0.0161
Esophagus	55	0.3627	0.6798	0.2215	0.6076	0.7333	0.6676	0.0065
Gastric body	244	0.5360	0.7641	0.3661	0.6752	0.7289	0.7229	0.0176

The analysis evaluates segmentation accuracy and structural quality across the three anatomical sites available in the validation split: Gastric Antrum, Esophagus, and Gastric Body

**Fig. 6:**
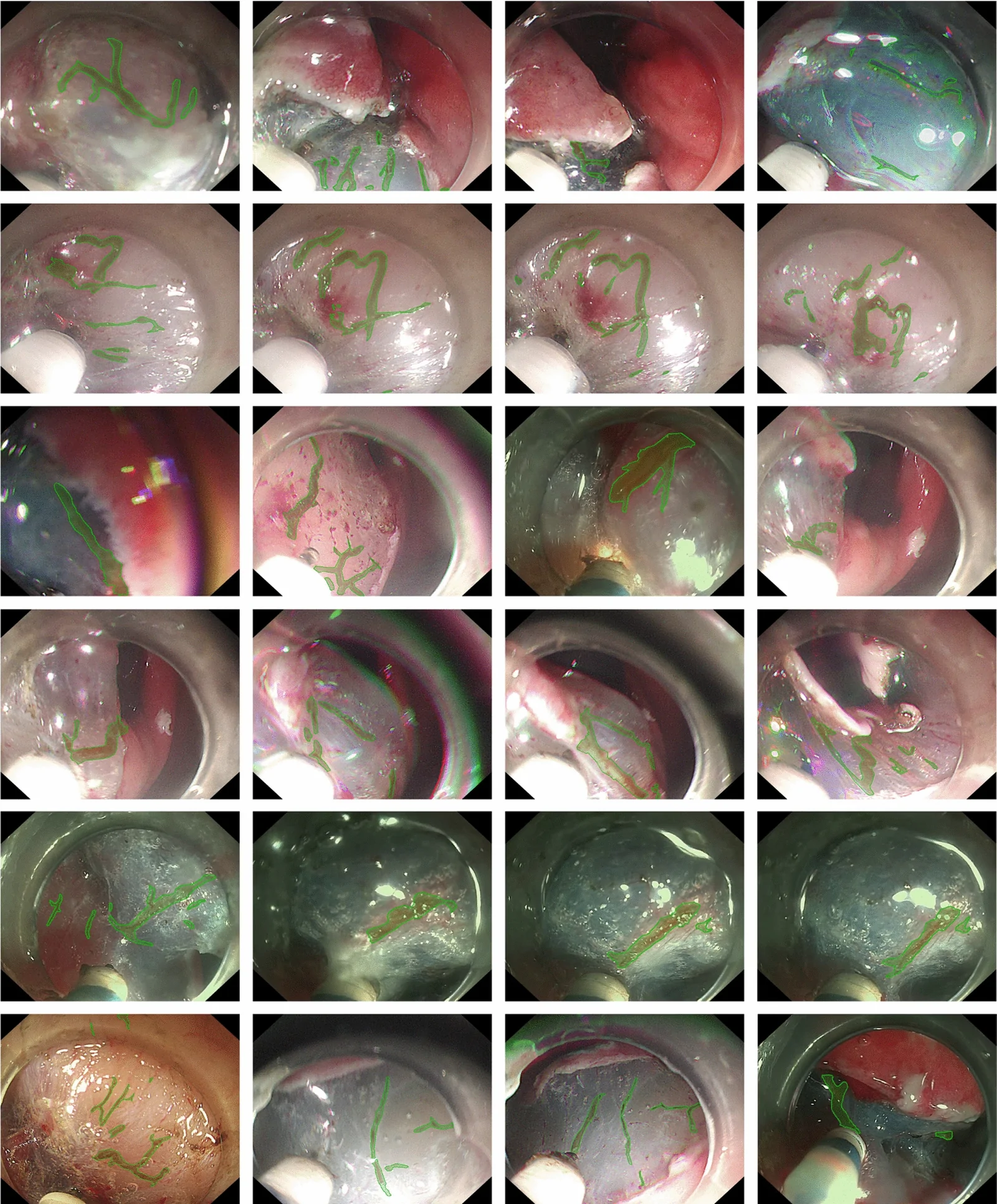
Segmentation visualization results using our ESD-VesNet across multiple sites. These results demonstrate the model’s ability to generalize to diverse patients and surgical scenarios

### Visualization of qualitative comparison

Figure 4 presents representative vessel segmentation results across different methods on challenging ESD frames. We compare our proposed approach against several state-of-the-art baselines, including U-Net, DeepLabv3+, TransUNet, and SAM-based variants. The visualization reveals distinct performance patterns across various surgical scenarios.

For frames containing clear, well-exposed vessels (Row 1), all methods achieve reasonable segmentation quality. However, our method demonstrates superior boundary precision and completeness in vessel delineation. In more challenging cases involving partial occlusions from bubbles or tissue debris (Row 2), baseline methods exhibit significant performance degradation. U-Net and DeepLabv3+ produce fragmented predictions with incomplete vessel structures, while TransUNet shows improved continuity but still suffers from boundary inaccuracies.

The most pronounced differences emerge in scenarios with extreme interference. When confronted with specular reflections and blood seepage (Rows 3–4), traditional methods generate substantial false positives, misclassifying mucosal folds and hemorrhagic regions as vessels. In contrast, our framework effectively suppresses these false detections while maintaining sensitivity to genuine vascular structures. The uncertainty-guided gating mechanism proves particularly effective in distinguishing confusable tissue patterns from true vessels.

Notably, vanilla SAM and prompt-free SAM variants demonstrate strong feature extraction capabilities but lack domain-specific optimization for ESD environments. Our integration of the evidential deep learning head with uncertainty-guided hard negative mining substantiallyenhances robustness against the dynamic noise patterns characteristic of ESD procedures, as evidenced by cleaner predictions with fewer spurious activations in anatomically implausible regions.

## Conclusion

The accurate segmentation of submucosal vessels is critical for safe ESD procedures. This work presents ESD-VesNet, a vessel segmentation framework for endoscopic submucosal dissection. By integrating SAM3 foundation model capabilities with evidential uncertainty quantification and FP-aware training with hard negative mining, our method achieves superior performance in detecting thin, camouflaged vessels while maintaining low false-positive rates under challenging intraoperative conditions.

The contributions include: (1) the ESD-Vessel dataset comprising 3109 frames with both positive vessel annotations and curated hard negatives, addressing a critical gap in surgical AI benchmarks; (2) a prompt-free SAM3-based architecture augmented with an evidential head for pixel-wise uncertainty estimation; and (3) an uncertainty-guided hard negative mining strategy that dynamically identifies and prioritizes challenging false-positive patterns during training.

Our validation results demonstrate that ESD-VesNet achieves Dice$_{FG}$ 52.11%, VDR 96.81%, and BG$_{FPR}$ 0.61%, substantially outperforming existing methods in balancing sensitivity and specificity. The framework’s robustness against bubbles, debris, reflections, and blood seepage positions it as a useful tool for pre-coagulation guidance, with potential to reduce intraoperative bleeding complications and improve patient safety in ESD procedures.

Future work should focus on multi-center validation, temporal modeling for video-based detection, and integration with real-time surgical guidance systems. Additionally, exploring lightweight architectures suitable for edge deployment and incorporating multi-modal information (e.g., narrow-band imaging) could further enhance clinical utility.

## Data Availability

https://github.com/chenming2214-alt/ESD-VesNet.

## Appendix

### More visualization

Figure 6 shows our ESD-VesNet segmentation visualization results for vessels on multiple sites, further illustrating clinical applicability.

## Code Availability

https://github.com/chenming2214-alt/ESD-VesNet.
